# Rapid diagnosis of acute HIV-1 infection cases: first real-world performance of point-of-care HIV-1 nucleic acid testing in China

**DOI:** 10.3389/fpubh.2025.1707432

**Published:** 2025-12-19

**Authors:** Mingzhu Niu, Xiaoqin Xu, Yuhua Shi, Xin Zhang, Peng Guan, Yu Wang, Lijuan Dong, Huichao Chen, Desheng Huang, Cong Jin

**Affiliations:** 1 Department of Epidemiology, School of Public Health, China Medical University, Shenyang, China; 2 Department of HIV/STD Prevention and Control, Jiangsu Provincial Center for Disease Prevention and Control, Nanjing, China; 3 Yunnan Center for Disease Control and Prevention, Kunming City, China; 4 National Key Laboratory of Intelligent Tracking and Forecasting for Infectious Diseases, National Center for AIDS/STD Control and Prevention, Chinese Center for Disease Control and Prevention, Beijing, China; 5 Department of Intelligent Computing, School of Intelligent Medicine, School of Fundamental Sciences, China Medical University, Shenyang, China

**Keywords:** acute HIV infection, point-of-care testing, viral load, diagnostic performance, turnaround time

## Abstract

**Background:**

Timely diagnosis of acute HIV infection (AHI) is critical for curbing transmission. However, the diagnostic window prior to antibody development limits the utility of conventional serological assays. Conventional laboratory-based nucleic acid testing (LABT) is often hindered by operational complexity and long turnaround times. Point-of-care nucleic acid testing (POCT) provides a rapid alternative.

**Methods:**

This multicenter cross-sectional study enrolled 1,829 adults from 13 cities in Jiangsu Province and Kunming, Yunnan Province, who had reactive screening results but negative or indeterminate Western blot outcomes. The diagnostic accuracy, quantitative correlation, and agreement of the POCT (Xpert® HIV-1 Viral Load point-of-care test) were compared with LABT using the Roche COBAS TaqMan v2.0 or Abbott RealTime HIV-1 assays, with follow-up seroconversion serving as the reference standard. Turnaround time from initial screening to NAT result reporting was compared using Kaplan–Meier analysis. Laboratory staff perceptions of POCT usability were evaluated via questionnaire.

**Results:**

Using follow-up seroconversion as a reference, POCT demonstrated a sensitivity of 98.87% and specificity of 100.00%, with almost perfect agreement with LABT (99.76%, Cohen’s *κ* = 0.994). Quantitative viral load results exhibited strong correlation between POCT and LABT (*r* = 0.89), with 94.64% of paired measurements falling within the limits of agreement. POCT significantly reduced the median time from initial screening to result reporting compared to LABT (7 vs. 14 days, *p* < 0.001) and increased the likelihood of results being returned within 7 days (Hazard Ratio = 2.29, *p* < 0.001). Most laboratory personnel reported ease of use (30/33, 90.9%) and a preference for the POCT workflow (21/33, 63.6%).

**Conclusion:**

The Xpert HIV-1 POCT provides a highly accurate and rapid alternative to LABT for diagnosing AHI in China. Its implementation can dramatically shorten diagnostic delays, potentially reducing loss to follow-up and onward transmission, thus offering significant value within the national HIV diagnostic framework.

## 1 Introduction

HIV-1 infection has led to 1.329 million people living with the virus and 0.474 millions of deaths by the middle of 2024 in China (1). Early diagnosis is essential for optimizing clinical outcomes and preventing onward transmission, particularly during acute HIV infection (AHI)—a stage characterized by extremely high viral loads and peak infectiousness (2, 3). AHI refers to the period between viral acquisition and the appearance of detectable antibodies, when transient viremia occurs but conventional antibody-based tests often fail to detect infection (4).

**Figure 1:**
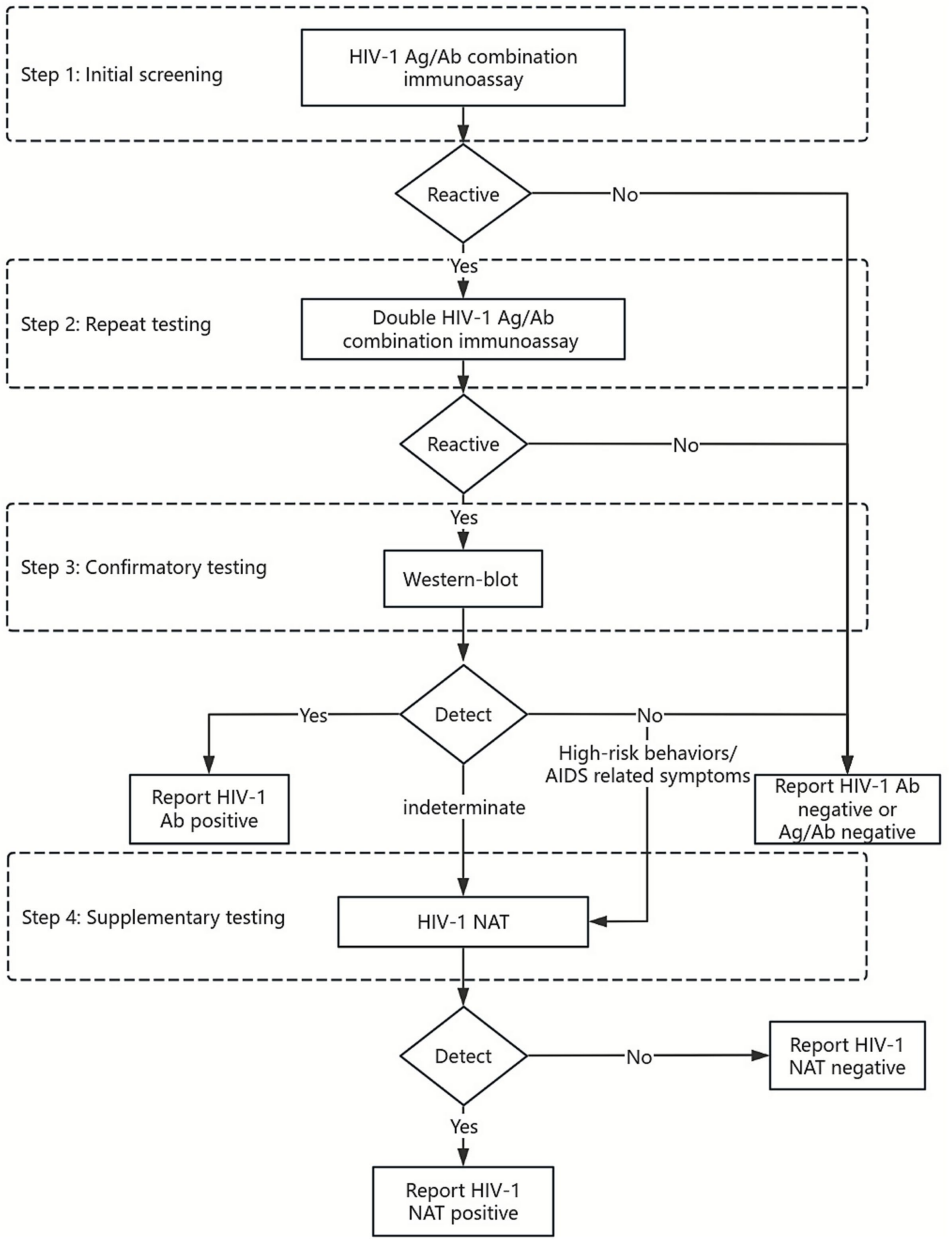
Algorithm for HIV diagnosis in China.

According to the updated 2020 Chinese Centers for Disease Control and Prevention diagnostic guidelines, HIV testing follows a multi-step algorithm. The initial screening employs an antigen–antibody immunoassay, with reactive samples undergoing repeat testing. Samples that remain reactive proceed to confirmatory testing, which has traditionally relied on the Western blot (WB) assay. However, due to its longer diagnostic window period, WB frequently yields false-negative or indeterminate results in AHI cases (5). In such situations, nucleic acid testing (NAT) is recommended as a supplementary test to establish diagnosis (Figure 1) (6).

Conventional laboratory-based NAT (LABT) requires advanced instruments, centralized facilities, and well-trained personnel, often leading to prolonged turnaround times and delayed diagnosis (7, 8). To address these limitations, point-of-care NAT (POCT) offers a promising alternative. In 2017, Xpert HIV-1 Viral Load (VL) assay (Cepheid; Sunnyvale, CA, USA) received WHO prequalification. POCT systems require minimal training, can be placed in primary health facilities, can be operated by staff in 90 min, and can reduce the turnaround time of results (9, 10). Beyond its established use in monitoring treatment efficacy, POCT has also been widely adopted for early diagnostic purposes, demonstrating high sensitivity and specificity in both acute HIV infection (AHI) detection and early infant diagnosis (11–16). This dual applicability underscores its potential value within China’s diagnostic framework, where timely identification of AHI remains a critical unmet need.

Previous evaluations of POCT have largely been conducted in sub-Saharan Africa under programmatic conditions (17, 18). To date, the potential of simplified and rapid POCT has not been evaluated on-site within the context of Chinese current diagnostic strategies and healthcare infrastructure. In this on-site study, we evaluated the diagnostic performance and operational feasibility of the POCT compared with LABT for diagnosing AHI in individuals with negative or indeterminate WB results. Additionally, we examined its impact on diagnostic turnaround time within the HIV testing algorithm.

## 2 Materials and methods

### 2.1 Study sites and study participants

This cross-sectional, multicenter study was conducted between August 2022 and December 2024 across 13 cities in Jiangsu Province (Nanjing, Suzhou, Wuxi, Changzhou, Zhenjiang, Nantong, Taizhou, Yangzhou, Yancheng, Huai’an, Suqian, Xuzhou, and Lianyungang) and Kunming in Yunnan Province. Eligible participants were adults (≥18 years old) who had not previously been diagnosed with HIV infection and had reactive results both in initial screening and repeat testing but negative or indeterminate results on WB. This inclusion criterion reflects the Chinese diagnostic algorithm, in which individuals with WB-negative or indeterminate results represent the most clinically relevant group in which POCT could add diagnostic value. Follow-up serologic testing was conducted to determine the final HIV infection status.

### 2.2 HIV-1 viral load quantitation

**Figure 2:**
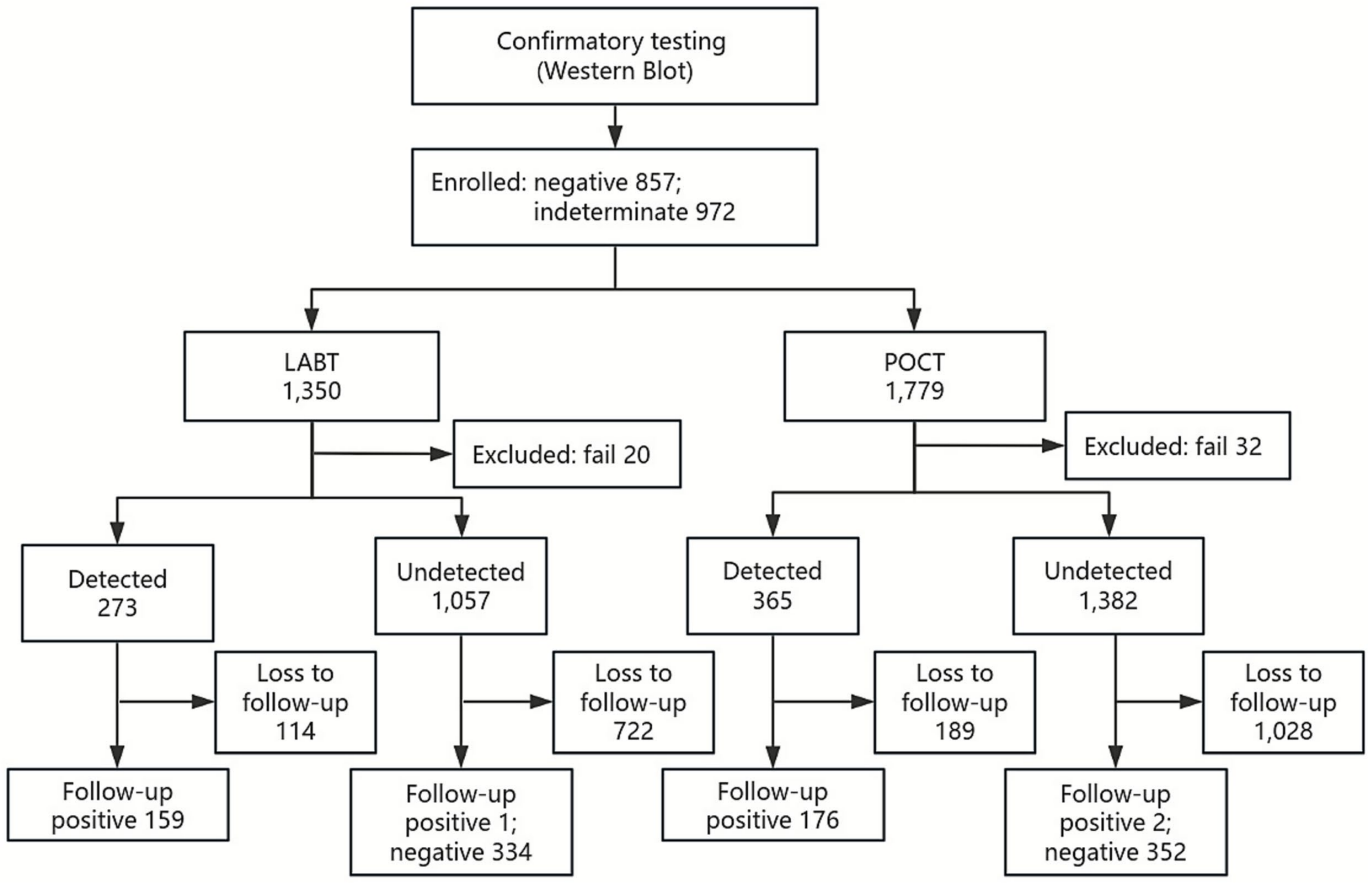
Flowchart of study design. POCT, point-of-care HIV-1 viral load test; LABT, laboratory-based viral load test.

A 10 mL whole blood sample was collected in EDTA tubes, and plasma was separated and divided into two aliquots for parallel testing. One aliquot was tested using POCT with the Xpert® HIV-1 Viral Load assay (Cepheid, Sunnyvale, CA, USA), while the other was tested using LABT with either the Roche COBAS TaqMan v2.0 (Roche assay) or the Abbott RealTime HIV-1 assay (Abbott assay), depending on site availability. The quantification range of the Roche assay is 20–10,000,000 copies/mL (1.3 log_10_ to 7.0 log_10_ copies/mL). The quantification range for both the Xpert assay and Abbott assay is 40–10,000,000 copies/mL (1.6 log_10_ to 7.0 log_10_ copies/mL). Because not all samples yielded sufficient plasma to support testing on both platforms, laboratory personnel at each study site selected the testing method according to the available plasma volume and local testing capacity. All assays were performed per manufacturers’ instructions. Study personnel, though experienced users, were trained for this purpose. Samples not processed immediately were stored at −80 °C. For each assay, the dates of sample collection, testing, and result report were recorded. A full flowchart of procedures is presented in Figure 2.

### 2.3 Usability evaluation

To assess usability and staff acceptance of the Xpert assay, a six-item questionnaire was developed, including four single-choice and two multiple-choice items. The questionnaire capturing steps to results, amount of ease of use, errors and overall opinion of the technology was filled in by each personnel based on their experience on the machine. The usability data was collected by 33 laboratory personnel from the 14 participating cities.

### 2.4 Statistical analysis

Diagnostic performance was evaluated by comparing the results of POCT and LABT against the follow-up WB outcomes. Qualitative results were categorized as “HIV-1 RNA detected” (≥limit of detection) or “undetected” (<limit of detection). Sensitivity, specificity, positive predictive value (PPV), and negative predictive were calculated. Assay concordance was assessed using percentage agreement and Cohen’s *κ*. Quantitative results were reported in log_10_ copies/mL. The Pearson correlation coefficient (*r*) and Bland–Altman analysis were used to assess the linear relationship and level of agreement between the quantitative results of the two assays.

Categorical variables were summarized as frequencies and percentages, and continuous variables were described using medians and interquartile ranges (IQRs). The *χ*^2^ test was used to compare the proportions of turnaround time between LABT and POCT. The Mann–Whitney U test was applied to assess differences in the median time from initial screening to NAT result reporting. Kaplan–Meier survival curves and log-rank tests were employed to compare time-to-result reporting distributions. For the analysis of 7-day reporting, results returned after day 7 were treated as right-censored at 7 days to ensure consistency and reproducibility of hazard ratio estimation. The failed test results were excluded from the analysis.

All statistical tests were two-sided, and *p* < 0.05 was considered to indicate statistical significance. Statistical analysis was conducted using IBM SPSS Statistics, version 27.0 (IBMCorp., Armonk, N.Y., USA) and GraphPad Prism software, version 10.1.2 (GraphPad Software, San Diego, CA, USA).

## 3 Results

### 3.1 Study population

Among individuals who showed reactivity in both the initial screening and repeat testing, a total of 1,829 participants with negative or indeterminate WB results were enrolled in this study. During follow-up, 541 participants were monitored, of whom 182 subsequently seroconverted, while 359 did not. A total of 1,350 samples underwent LABT, 20 (1.48%) generated failed results and were excluded, leaving 1,330 valid test results (273 cases were detected, and 1,057 were not). In parallel, 1,779 samples underwent POCT, 32 (1.80%) generated failed results and were excluded, leaving a total of 1,747 samples with valid test results (365 cases were detected, and 1,382 were not).

### 3.2 Diagnosis performance of the POCT and LABT

Using WB follow-up results as the reference standard, diagnostic performance was assessed for POCT (*n* = 524) and LABT (*n* = 489). Among the cases analyzed, two seroconversion cases were undetected by POCT, resulting in a sensitivity of 98.87% (95% Confidence Interval [CI]: 96.09–99.87%) and specificity of 100.00% (95% CI: 99.15–100.00%). The PPV of the POCT was 100.00% (95% CI: 98.32–100.00%), and the NPV was 99.43% (95% CI: 98.16–99.91%). LABT failed to detected one seroconversion case, resulting in a sensitivity of 99.37% (95% CI: 96.23–100.00%) and a specificity of 100.00% (95% CI: 98.79–100.00%). The PPV of the LABT was 100.00% (95% CI: 97.89–100.00%), and the NPV was 99.70% (95% CI: 98.18–100.00%) (Table 1).

**Table 1:** Diagnostic performance of POCT and LABT compared to follow-up Western blot results.

Testing results		WB			Sensitivity% (95% CI)	Specificity% (95% CI)	PPV% (95% CI)	NPV% (95% CI)
		Seroconversion	Non-seroconversion	Total				
POCT	Detected	175	0	175	98.87 (96.09–99.87)	100.00 (99.15–100.00)	100.00 (98.32–100.00)	99.43 (98.16–99.91)
	Undetected	2	347	349				
	Total	177	347	524				
LABT	Detected	158	0	158	99.37 (96.23–100.00)	100.00 (98.79–100.00)	100.00 (97.89–100.00)	99.70 (98.18–100.00)
	Undetected	1	330	331				
	Total	159	330	489				

WB, Western blot; PPV, positive predictive value; NPV, negative predictive value; POCT, point-of-care HIV-1 viral load test; LABT, laboratory-based viral load test; CI, confidence interval.

A total of 419 samples were tested in parallel by POCT and LABT, including 334 used the Roche assay and 85 with the Abbott assay. The overall agreement between LABT and POCT was 99.76% (95% CI: 99.29–100.00%), with a Cohen’s *κ* = 0.994 (95% CI: 0.985–1.000; Table 2). The agreement with the Roche assay was 99.70% (95% CI: 99.11–100.00%, κ = 0.989, 95% CI: 0.960–1.000). The agreement with the Abbott assay was 100.00% (95% CI: 78.75–100.00%, Cohen’s *κ* = 1.000, 95% CI: 0.899–1.000) (Appendix 1 Table 1).

**Table 2:** Agreement between POCT and LABT results in parallel-tested samples.

POCT	LABT			Agreement% (95% CI)	Kappa (95% CI)
	Detected	Undetected	Total		
Detected	112	0	112	99.76 (98.10–100.00)	0.994 (0.985–1.000)
Undetected	1	306	307		
Total	113	306	419		

POCT, point-of-care HIV-1 viral load test; LABT, laboratory-based viral load test.

### 3.3 Correlation and agreement between POCT and LABT

**Figure 3:**
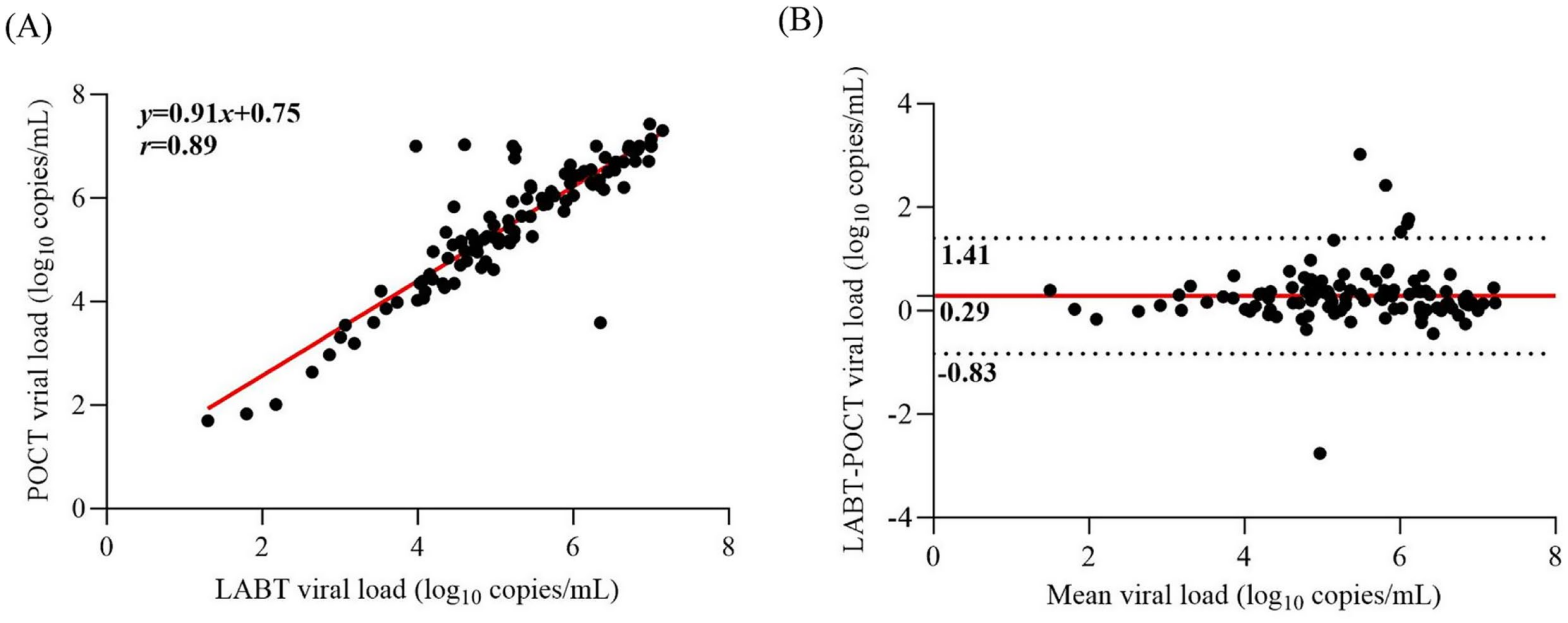
Pearson correlation and Bland–Altman analyses between the POCT and the LABT. **(A)** Pearson correlation plot between two assays of all analyzable 112 samples (Pearson *r* = 0.89, *p* < 0.001). **(B)** Bland–Altman scatter plot between two assays. The x axe shows the averages of two assays, and the y axe shows the bias between two assays. Bias was defined as the LABT log_10_ copies/mL of viral load minus the POCT log_10_ copies/mL of viral load. The red solid line indicates the mean bias and the dashed lines show the limits of statistically acceptable bias defined as the mean bias ± 1.96 standard deviations of bias. POCT, Xpert point-of-care HIV-1 viral load test; LABT, laboratory-based viral load test.

Quantitative performance was evaluated in 112 samples with quantifiable results from both assays. The Pearson correlation coefficient between POCT and LABT was 0.89 (*p* < 0.001; Figure 3A). Bland–Altman analysis showed correlation with a mean bias of 0.29 log_10_ copies/mL, with 94.64% (106/112) of paired VLs falling within the 95% limits of agreement (−0.83 to 1.41 log_10_ copies/mL; Figure 3B).

Further analysis compared the Xpert assay with the Roche assay (*n* = 93) and Abbott assay (*n* = 19). The Pearson correlation coefficient between the Xpert assay and the Roche assay was 0.92 (*p* < 0.001; Appendix 2 Figure 2A). Bland–Altman analysis showed a mean bias of 0.23 log_10_ copies/mL, with 95.70% (89/93) falling within the 95% limits of agreement (−0.72 to 1.18 log_10_ copies/mL; Appendix 2 Figure 2B). Between Xpert and Abbott, the correlation was 0.85 (*p* < 0.001; Appendix 2 Figure 2C). Bland–Altman analysis showed a mean bias of −0.43 log_10_ copies/mL, with 89.47% (17/19) falling within the 95% limits of agreement (−1.05 to 2.21 log_10_ copies/mL; Appendix 2 Figure 2D).

### 3.4 Turnaround time and reporting efficiency

**Figure 4:**
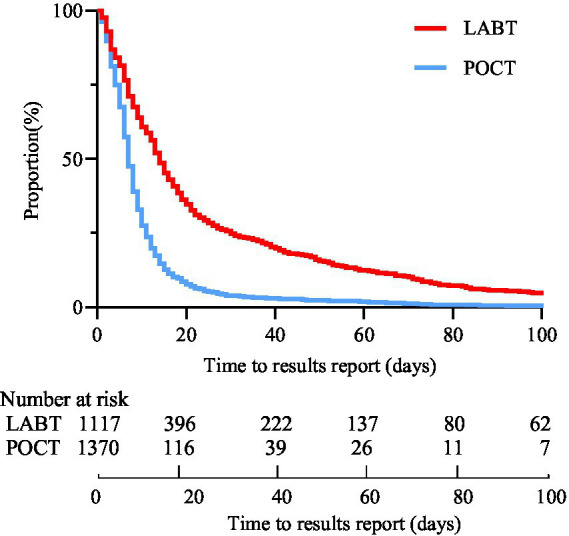
Kaplan–Meier curve comparing time from initial screening to NAT result reporting between POCT and LABT. POCT, point-of-care HIV-1 testing; LABT, laboratory-based testing.

This study found a significant improvement in service delivery outcomes with POCT relative to LABT. The median turnaround time from initial screening to supplementary testing was improved from 14 days (IQRs: 6–29) with LABT to 7 days (IQRs: 4–11) with POCT (*p* < 0.001). The median turnaround time of NAT was same day (IQR: 0–1) with POCT in comparison to 6 days (IQR: 1–23) with LABT (*p* < 0.001). The proportion of valid NAT results reported within 7 days was significantly higher with POCT (731/1,370 [53.36%]) compared to LABT (342/1,117 [30.62%]; *p* < 0.001; Table 3). The Kaplan–Meier survival curves show that POCT achieves a much faster decline in the probability of not receiving results, indicating more rapid return of results compared to LABT (Figure 4). The likelihood of early report of NAT results (within 7 days) was significantly higher with POCT compared to LABT (hazard ratio 2.29, 95% CI: 2.03–2.59; *p* < 0.001). In addition, the median time from initial screening to follow-up seroconversion for all WB negative or uncertain participants was 39 days (IQR: 25–54).

**Table 3:** Comparison of service delivery outcomes between POCT and LABT.

Variables	POCT	LABT	*p* value
Number of tests done	1,779	1,350	–
Time from initial screening to NAT results report, days	7 (4–11)	14 (6–29)	<0.001
Initial screening, days	0 (0–0)	0 (0–0)	1.000
Repeat testing, days	3 (0–7)	3 (0–8)	0.296
Confirmatory testing, days	1 (0–5)	1 (0–5)	0.319
Supplementary testing, days	0 (0–1)	6 (1–23)	<0.001
The proportion of NAT results reported within 7 days	53.36% (731/1,370^#^)	30.62% (342/1,117^#^)	<0.001
The proportion of NAT results reported within 15 days	74.53% (1,021/1,370^#^)	55.86% (624/1,117^#^)	<0.001
The proportion of NAT results reported within 30 days	96.13% (1,317/1,370^#^)	75.92% (848/1,117^#^)	<0.001

Data are median (IQR) or % (*n*). ^#^The denominator is the number of tests recorded on the date of detection. POCT, point-of-care HIV-1 nucleic acid testing; LABT, laboratory-based HIV-1 nucleic acid testing; NAT, nucleic acid testing.

### 3.5 Evaluation of usability characteristics

**Figure 5:**
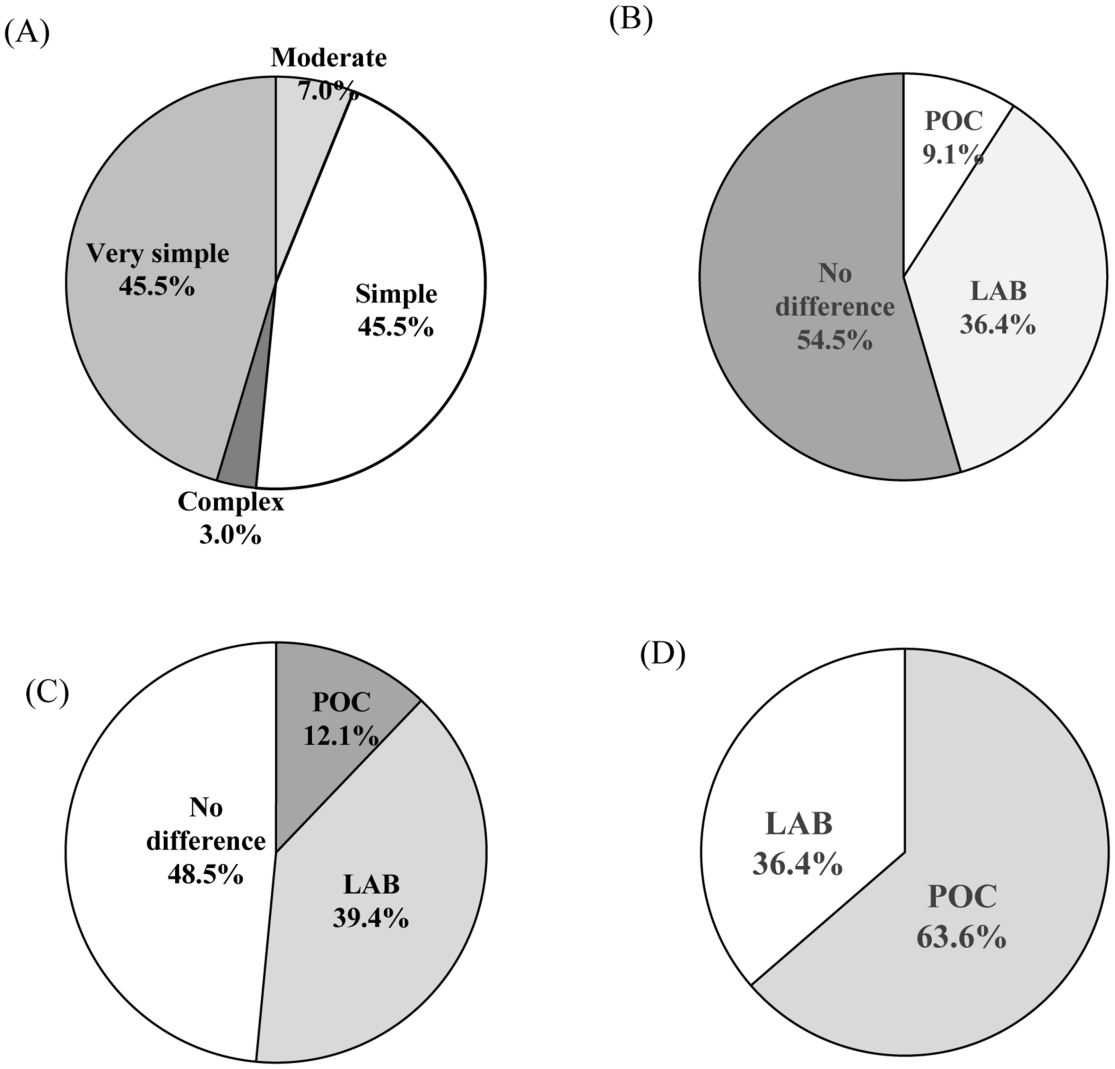
Laboratory staff perceptions of POCT versus LABT platforms. **(A)** Perceived simplicity of POCT workflow; **(B)** Equipment most susceptible to errors during sample loading; **(C)** Equipment most susceptible to errors during reagent handling; **(D)** Preferred testing platforms for routine use. POC, Xpert point-of-care HIV-1 viral load assay; LAB, laboratory-based viral load assay.

Among 33 laboratory staffs from 14 participating cities, 54.5% (18/33) had more than 5 years of experience in VL testing. A majority (90.9%, 30/33) reported the POCT platform as easy to use with a simple workflow (Figure 5A). While 54.5% (18/33) reported no significant differences in sample loading error rates between platforms (Figure 5B), 39.4% (13/33) noted a higher frequency of reagent-handling errors with LABT (Figure 5C). Overall, 63.6% (21/33) preferred the POCT platform (Figure 5D), citing ease of operation, shorter turnaround time, and compact device design. Reported challenges with the Xpert assay included high maintenance requirements, elevated reagent costs, and occasional module malfunctions.

## 4 Discussion

In recent years, HIV NAT has expanded beyond its traditional role in monitoring treatment efficacy and is now also recommended for diagnosing HIV infection, particularly for identifying acute infected individuals. This shift has led to increasing demand for NAT-based services (19). Consequently, there is an urgent need for accessible, user-friendly HIV-1 POCT platforms that can replace or complement conventional NAT methods, which are typically performed in centralized laboratories. Among current available approaches, the Xpert assay represents the most promising candidate due to its simplicity, ease of use, and rapid turnaround time (20).

Our findings are consistent with previous evaluations of the Xpert HIV-1 POCT in African programmatic settings, which also demonstrated excellent diagnostic accuracy and shorter turnaround times compared with centralized laboratory testing. However, unlike those studies conducted in high-prevalence, resource-limited contexts, our multicenter study provides novel, real-world evidence on the feasibility of integrating POCT into China’s diagnostic framework. In contrast to prior work that primarily assessed analytical performance under laboratory conditions in Europe and North America, our evaluation highlights the operational benefits and challenges of POCT when applied within the national algorithm. Although the quantitative correlation observed in our study was slightly lower than in earlier laboratory-based reports (21, 22), this difference likely reflects heterogeneity between laboratory evaluations and real-world field conditions. Importantly, the comparison in our study refers specifically to results obtained from POCT relative to LABT, rather than direct analytical equivalence of the assays themselves. Conducting the study in a real-world setting introduced additional variability in testing environments, including differences in conditions, methodologies, timing, and personnel, thereby offering unique insights into practical challenges (23). By situating the evaluation within actual clinical practice, our study offers valuable insights into the practical factors that influence POCT performance and underscores the importance of further assessments across diverse settings to ensure accuracy, reliability, and applicability in routine diagnostics.

Two atypical cases encountered in this study highlight the complexity of interpreting HIV NAT results in clinical practice. In one case, both POCT and LABT failed to detect viral load due to ongoing antiretroviral therapy, despite subsequent seroconversion confirmed by WB. In another case, POCT failed to detect VL, whereas LABT (Roche assay) reported 7,280 copies/mL. This discrepancy likely reflects assay design: the Roche assay targets both LTR and gag regions, while the Xpert assay targets only LTR, potentially leading to mismatches (24). Genotypic testing identified infection with the HIV-1 *CRF 07_BC* subtype, one of the major circulating subtypes in China. Subsequent repeat testing using a dual-target Cepheid assay, which is not currently approved by the CFDA, yielded a positive result. These cases highlight the complexity of HIV diagnostic practices in real-world settings and underscore the need for cautious interpretation of negative HIV nucleic acid results, particularly in individuals with high-risk behaviors or clinical indicators of infection. Clinical context, treatment history, and viral subtype variation must be carefully considered when results are discordant.

Loss to follow-up remains a substantial challenge in China, where many individuals identified through initial screening are not retained due to prolonged Western blot confirmation protocols for indeterminate cases (25). Notably, it is estimated that 10–50% of HIV transmission events may be attributed to AHI (26). In the current national diagnostic algorithm, WB is still the primary confirmatory tool; however, its long window period often results in false-negative or indeterminate outcomes, delaying timely diagnosis and treatment. HIV NAT is anticipated to play a crucial role in mitigating this issue. This study demonstrated that POCT significantly decreased turnaround time for result reporting compared to LABT (7 *vs*. 14) and follow-up WB (7 *vs*. 39). These findings corroborate previous research and underscore the benefits of POCT in enhancing diagnostic efficiency and reducing turnaround time (27, 28). Additionally, the use of POCT is likely to further facilitate the utilization and accessibility of HIV NAT in China. Most regions in China exhibit low HIV-1 prevalence, leading to a limited routine demand for testing. The batch processing characteristic of conventional LABT frequently results in extended sample waiting times within centralized laboratories and limiting its practical application in primary health care institution. Conversely, POCT offers flexible throughput and facilitates real-time, on-demand testing, significantly reducing sample waiting times to enable immediate testing (29). This approach aligns more effectively with the current epidemiological context in China.

The Xpert assay exhibited comparable error rates and ease of use, making it well-suited for widespread implementation in China. In our study, we observed that the error rate of both LABT and POCT testing was less than 2%. The error rate of the Xpert assay (1.80%) was slightly higher than that of conventional testing methods (1.48%). For POCT, the errors or invalid results observed were attributed to insufficient sample volumes, quality control issues of the test kits. These error causes have also been reported in previous studies (30, 31). Moreover, repeated detection can further reduce the error rate (32). Among the 33 laboratory personnel who participated in the questionnaire survey, 30 (90.9%) reported that the POCT was user-friendly and had a straightforward operational process. 63.6% of participants expressed a preference of POCT for routine use, suggesting that the compact instrument size and simplified workflow of the Xpert assay effectively meet the needs of primary healthcare laboratories. Therefore, during the process of promoting its use, it remains essential to strengthen reagent quality control and personnel training to ensure the high accuracy and reliability of the Xpert assay across diverse clinical settings (33).

Our study had several limitations. First, we did not assess the impact of POCT on the time to treatment initiation, as our evaluation focused primarily on diagnostic timeliness, accuracy, and operational feasibility. Second, we did not include cost-effectiveness analyses, which are essential for understanding the broader implications of implementing POCT in routine practice. Third, the significant loss to follow-up may limit the generalizability of the findings. While the follow-up group showed high diagnostic accuracy, the characteristics of the lost-to-follow-up participants are unknown, and this attrition could affect the broader applicability of the results to the entire enrolled population.

## 5 Conclusion

The Xpert assay offers a practical and accurate alternative to conventional laboratory testing, with NAT results comparable to high-throughput platforms. By enabling rapid, on-site detection and earlier identification of AHI, POCT shortens turnaround times and mitigates challenges of patient attrition, ongoing transmission, and resource burden associated with prolonged follow-up of WB-negative or indeterminate cases. Ensuring robust reagent quality control and adequate personnel training will be critical to sustaining its reliability in routine practice.

## Data Availability

The original contributions presented in the study are included in the article; further inquiries can be directed to the corresponding author.
