# Supplementary material for: Rapid diagnosis of acute HIV-1 infection cases: first real-world performance of point-of-care HIV-1 nucleic acid testing in China
Source: Front Public Health. 2025 Dec 19;13:1707432. doi: 10.3389/fpubh.2025.1707432 (PMC12757961; doi:10.3389/fpubh.2025.1707432)
Supplement: Supplementary file 1 [file Supplementary_file_1.docx]

**Appendix 1**

**Table 1 Comparison of POCT diagnosis performance against Roche and Abbott assays.**

| **LABT** | | **POCT** | | | **Agreement% (95% CI)** | **Kappa (95% CI)** |
| --- | --- | --- | --- | --- | --- | --- |
|  |  | Detected | Undetected | Total |  |  |
| Roche | Detected | 93 | 1 | 94 | 99.70 (99.11-100.00) | 0.989 (0.960-1.000) |
|  | Undetected | 0 | 240 | 240 |  |  |
|  | Total | 93 | 241 | 334 |  |  |
|  |  |  |  |  |  |  |
| Abbott | Detected | 19 | 0 | 19 | 100.00 (78.75-100.00) | 1.000 (0.899-1.00) |
|  | Undetected | 0 | 66 | 66 |  |  |
|  | Total | 19 | 66 | 85 |  |  |

**Appendix 2**


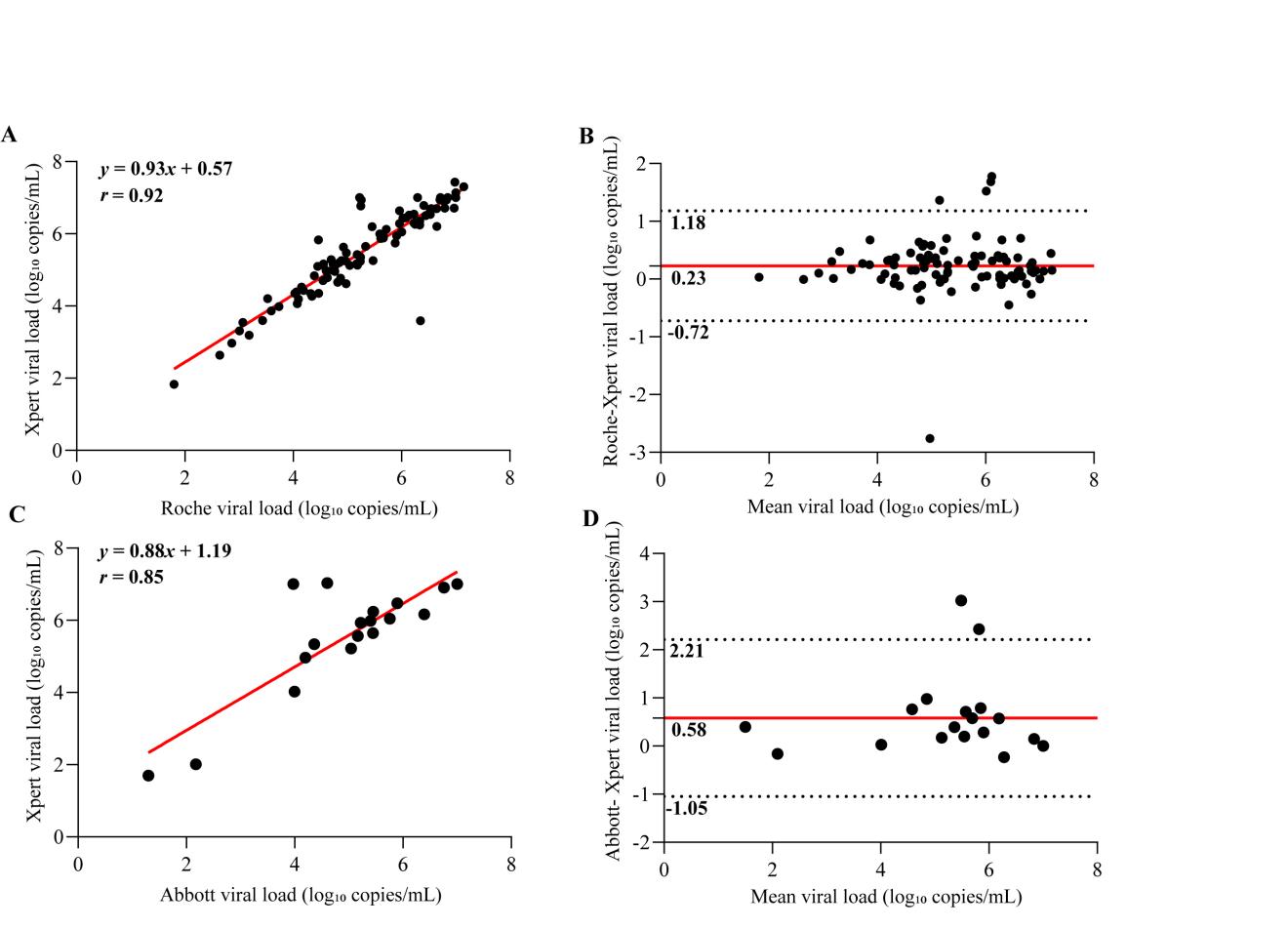


**Figure 2 Pearson correlation and Bland-Altman analyses between the POCT and the LABT.**

(A) Pearson correlation between the Xpert assay and the Roche assay. (B) Bland-Altman plot of Xpert vs. Roche assay. (C) Pearson correlation between the Xpert assay and the Abbot assay. (D) Bland-Altman plot of Xpert vs. Abbott assay. In Bland-Altman plots, the x-axis shows the average of the two assays, and the y-axis shows the bias between them. The red solid line represents the mean bias, and the dashed lines indicate the limits of agreement (mean bias±1.96 standard deviations).
